# Massive Life-Threatening Saddle Pulmonary Embolism in a Healthy 28-Year-Old

**DOI:** 10.7759/cureus.37789

**Published:** 2023-04-18

**Authors:** Prachi B Patel, Suresh Ramamurthy

**Affiliations:** 1 Department of Cardiology, Philadelphia College of Osteopathic Medicine, Suwanee, USA; 2 Department of Cardiology, Wellstar North Fulton Hospital, Roswell, USA

**Keywords:** right ventricular shock, cor pulmonale, thrombectomy, hypercoagulable, deep vein thrombosis (dvt), out of proportion pulmonary hypertension, saddle pulmonary embolism

## Abstract

When Virchow’s triad is disrupted, a deep vein thrombosis (DVT) can often occur and progress into a pulmonary embolism, and in rare cases, a saddle pulmonary embolism. This 28-year-old male patient showed up at the emergency department (ED) with shortness of breath, chest palpitations, and right calf pain. Additional imaging showed a massive saddle pulmonary embolism, and he was taken to immediate right femoral catheterization for thrombectomy. Though this patient presents with no known risk factors in his history or workup, he stretches the predefined boundaries with his cavalier presentation.

## Introduction

Deep vein thromboses (DVTs) fall under the category for the third most common cause of cardiovascular death after heart attacks and strokes. An annual estimated rate of 200,000 people in the United States get DVTs each year and of that amount, 50,000 progress to pulmonary embolism. DVT is when a clot forms from a combination of trauma, immobility, genetic mutations, or other factors [1]. High-risk factors include those that are older, are obese, and/or have underlying malignancies [2]. The median age for DVTs found in recent studies is 69 years of age with a body mass index of 33 kg/m2 [3].

In roughly 55% of patients, DVTs are found in the proximal femoral vein and present with pain or persistent calf cramping, swelling, and redness [1,4]. When DVTs’ clot breaks off from a vessel, they can travel to the pulmonary arteries and cause an embolism. Typical symptoms are shortness of breath, chest pain exacerbated by deep inhalation, lightheadedness, dyspnea at rest, anxiety, and syncope [4,5].

Saddle pulmonary emboli are a type of venous thromboembolism located at the bifurcation of the pulmonary artery with the potential to cause cardiogenic shock and death [3]. These conditions call for urgent treatment via a catheter-driven thrombectomy or thrombolysis. The clotting and transit process is quick; therefore, immediate action is necessary. However, diagnosis and treatment can often get delayed due to the potential asymptomatic presentation.

## Case presentation

A 28-year-old male cab driver with a history of asthma presented to the emergency department (ED) in September 2022 with shortness of breath and chest palpitations for three days as well as right calf pain for one day. The patient was tachycardic (a heart rate of 150 beats per minute), hypertensive (156/86), mildly hypoxemic (SpO2 of 94%), and tachypneic (a respiratory rate of 35 breaths per minute). He occasionally drank alcohol and had never smoked before. The patient had been admitted to the ED a month prior with sharp chest pains that radiated to the upper back and worsened with deep breaths and while laying down, but he was discharged after a clean chest X-ray, normal electrocardiogram showing no signs of ischemia or STEMI, and negative troponin levels.

The patient’s sedentary occupation caused suspicion of a DVT that was confirmed with a bilateral lower extremity venous duplex and D-dimer. The right popliteal vein was partially compressive, representing a semi-occlusive right popliteal DVT. The D-dimer was also well above the normal range (Table 1).

**Figure 1:**
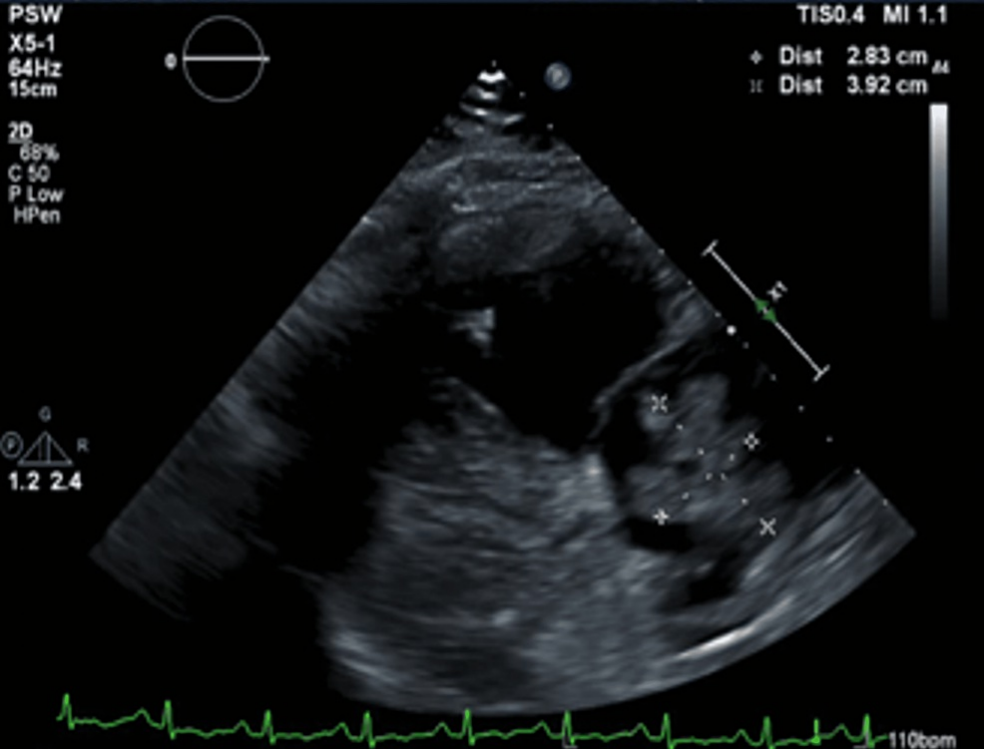
Transthoracic echocardiogram showing 2.83 cm × 3.92 cm right atrial thrombus

**Figure 2:**
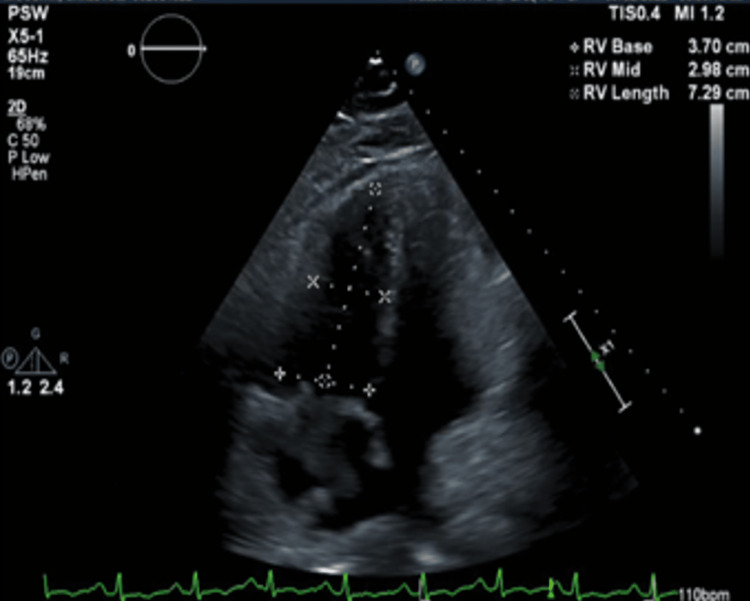
Transthoracic echocardiogram of apical four-chamber view showing right ventricular dilation

**Figure 3:**
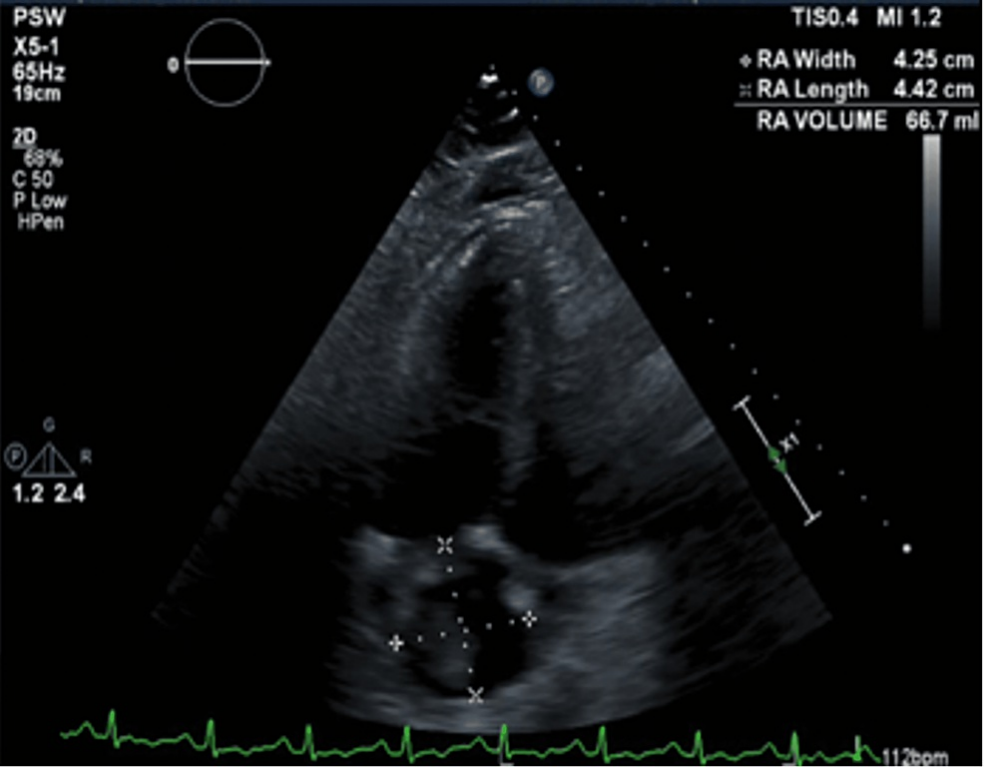
Transthoracic echocardiogram of apical four-chamber view showing right atrial dilation

The echocardiogram showed a large clot in the right atrium and ventricle in transit. They were moderately dilated with the right atria having a large protruding, echogenic, mobile 2.83 cm × 3.92 cm thrombus in the cavity, the right ventricle moderately dilated, and systolic function severely reduced (Figures 1-3). Furthermore, the central venous pressure was high at 15 mmHg in the inferior vena cava (normal range: 8-12 mmHg).

**Table 1:** Laboratory values

Laboratory test	Value	Reference range
D-dimer	58,995 ng/mL	0-500 ng/mL
Troponin T, high sensitivity	77 ng/L	0-15 ng/L
PBNP N terminal	3,193 pg/mL	0-300 pg/mL

**Figure 4:**
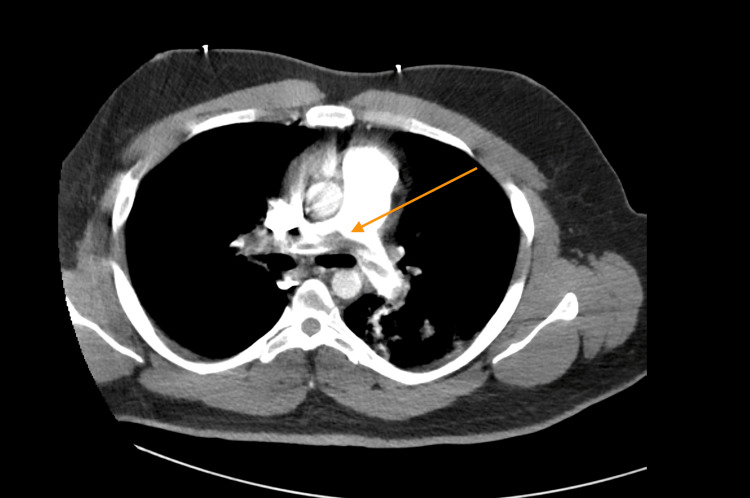
CT angiogram showing saddle embolus in pulmonary arteries

CT angiogram showed a small pulmonary embolic filling defect within bilateral main, segmental, and subsegmental pulmonary arteries. There was moderate to severe right-sided heart strain with a significant increase in the right-to-left heart size ratio. There were also infarcts in the left lung lower lobe (Figure 4).

The blood work presented with elevated troponin and brain natriuretic peptide provided further evidence of a heart strain, and thus, the patient was scheduled for a pulmonary thrombectomy (Table 1). The patient tolerated this procedure well, and a post-surgery echocardiogram showed that the clot burden in the right atrium had significantly improved. He was discharged on anticoagulation from the hospital without any complications and scheduled for a follow-up in three days. As the patient tested negative for COVID-19 and did not have any concerns for malignancy or a family history of clot or blood disorders, his presentation sets this case apart from the traditional saddle pulmonary embolism depictions specifically in populations this young.

## Discussion

**Figure 5:**
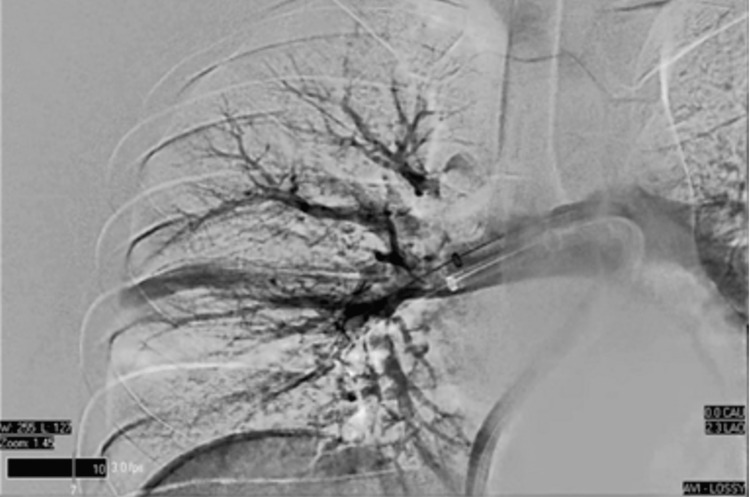
Ultrasound-guided right femoral vein access catheterization of the right lung

**Figure 6:**
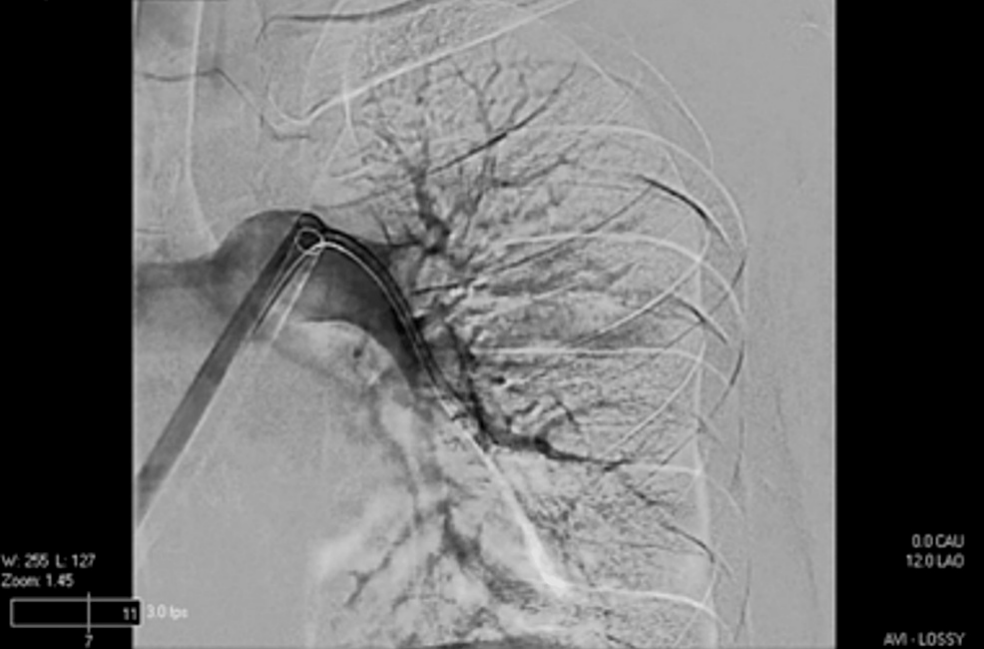
Ultrasound-guided right femoral vein access catheterization of the left lung

**Figure 7:**
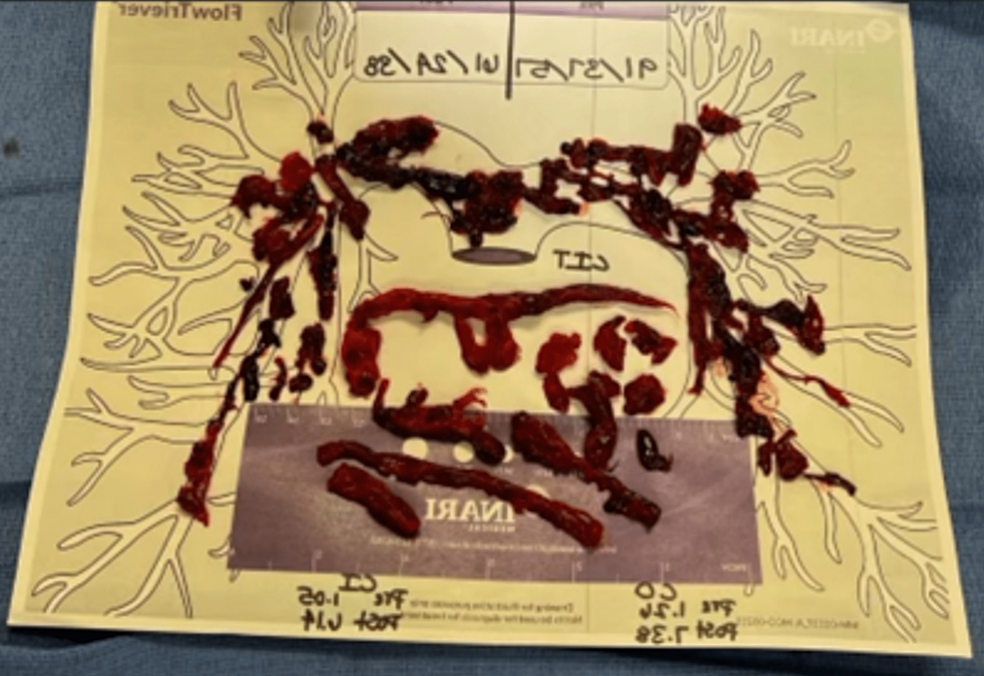
Clots retrieved from pulmonary arteries, suprarenal inferior vena cava, right atrium, and right ventricular thrombectomy

Saddle pulmonary embolism generally affects the older population unlike in this young patient’s case. An ultrasound-guided right femoral vein access catheterization was done where massive clots were found in the heart, pulmonary arteries, and lungs (Figures 5, 6) leading to severe pulmonary hypertension, cor pulmonale, and right ventricular shock. The patient also went through a bilateral pulmonary, suprarenal inferior vena cava, and right atrial and ventricular thrombectomy producing massive clots that were retrieved and modeled (Figure 7).

There are many saddle pulmonary embolism presentations unique to this patient. He had no underlying hypercoagulable or traumatic conditions that would skew Virchow’s triad. Though this 28-year-old withstood long hours of driving as an Uber driver, it is difficult to understand this one factor causing a massive saddle pulmonary embolism. About 68% of saddle embolic patients also have increased right atrial pressures (>5 mm Hg), whereas this patient did not present the same way [3]. Studies show that patients who survived hospitalization with a saddle pulmonary embolism had a PBNP of 2,394 +/− 532 pg/mL (Table 1). In this case, the patient's PBNP does not correlate with previous studies as this value is far beyond the average researched PBNP for patients with saddle pulmonary emboli [3].

What sets this patient apart is that he has no comorbidities or significant risk factors that could account for his condition. Furthermore, his hypercoagulability workup after surgery showed that he was negative for factors II and V, had <2.0 GPL cardiolipin AB, and had <0.2 u/mL B2 glycoprotein, thus ruling out lupus and antiphospholipid syndrome. This asymptomatic patient was sent home from a previous ED visit after a normal electrocardiogram, negative chest X-ray, and within-range laboratory values that ruled out life-threatening causes of chest pain such as acute coronary syndrome, aortic dissection, and others. Though subtle, his symptoms could have been the start of his saddle pulmonary embolism.

## Conclusions

Though saddle pulmonary embolisms are rare, it is important to further promote research in this field and awareness to those with similar occupations due to the significant morbidity of this condition. Saddle emboli are often silent before they progress to acute cardiogenic shock or leave survivors with chronic pulmonary hypertension. This disease can also steer away from known risk factors as seen in this patient. It is rare for a 28-year-old to get this with negligible previous malignancy, trauma, surgical, and genetic history. This emphasizes the need for continued exploration such as further hypercoagulability workup, specifically proteins C and S, as well as genetic breakdown for pulmonary embolism risk factors. More research will help define the underlying etiologies and presentations for saddle pulmonary emboli to help initiate earlier diagnosis and prevent increased mortality and post-thrombotic comorbidities.
